# Generic switch-over during ontogenesis in *Dimorphacanthella* gen. n. (Collembola, Isotomidae) with barcoding evidence

**DOI:** 10.3897/zookeys.73.839

**Published:** 2010-12-29

**Authors:** Mikhail B. Potapov, Yun Bu, Cheng-Wang Huang, Yan Gao, Yun-Xia Luan

**Affiliations:** 1 Moscow State Pedagogical University, Kibalchich str., 6, korp. 5, Moscow, 129278 Russia; 2 Institute of Plant Physiology and Ecology, Shanghai Institutes for Biological Sciences, Chinese Academy of Sciences, Shanghai, 200032 China

**Keywords:** Anal spines, New genus, Chinese Collembola, Barcoding analysis

## Abstract

A new genus Dimorphacanthella is established for Tetracanthella anommatos Chen and Yin, 1984 and Dimorphacanthella mediaseta **sp. n.** from China. The new genus exhibits an unusual metamorphosis: small juveniles, previously called Uzelia anommatos Yue & Yin, 1999 get the second pair of anal spines resulted from moulting and become “Tetracanthella”. Species identity of forms with two and four anal spines is proved by barcoding analysis. The derivation of anal spines is compared among genera having four anal spines.

## Introduction

With this paper we continue our study of Collembola of family Isotomidae from China (Gao et al. 2009; Gao and Potapov in press; Huang et al. 2009, 2010). After recent revisions of the genus Tetracanthella Schött, 1891 one species Tetracanthella anommatos Chen and Yin, 1984 remained curious (Deharveng 1987; Potapov 2001). Except possessing four anal spines, this odd species has no pigment or ocelli and exhibits an uncommon number and arrangement of sensilla on the body (Chen and Yin 1984). Its generic position has not been fully decided. During our study of the family Isotomidae of China we had an opportunity to collect this species from type (Shanghai) and other localities. In several populations Tetracanthella anommatos was mixed with another unusual blind form which was identified by us as Uzelia anommatos Yue & Yin, 1999, which was also described from Shanghai area. The latter one shares several important characters with Tetracanthella anommatos and was always recorded by us in the juvenile form. This led us to re-describe these two species using the modern morphological knowledge and to test their independence by barcoding analysis. In our collection from Northwest China (Ningxia Province), another species closely related to Tetracanthella anommatos was found. In this paper we establish a new genus, having uncommon metamorphosis during ontogenesis, re-describe Tetracanthella anommatos, and describe a new species.

Abbreviations: Abd. I–VIabdominal segments; Ant. I–IVantennal segments; AOantennal organ of antennal segment 3; bmsbasal microsensillum on antennal segments; Md, Mldorsal and lateral macrochaeta; msmicrosensillum; PAOpostantennal organ; ssensillum; Th. I–IIIthoracic segments; Ti. I–IIItibiotarsi of 1–3 pairs of legs.

## Taxonomy

### Dimorphacanthella gen. n.

urn:lsid:zoobank.org:act:EFBFAE0B-EC1C-4F41-90A8-6CAFC06CA6CC

#### Description.

**Figures 1–4.**
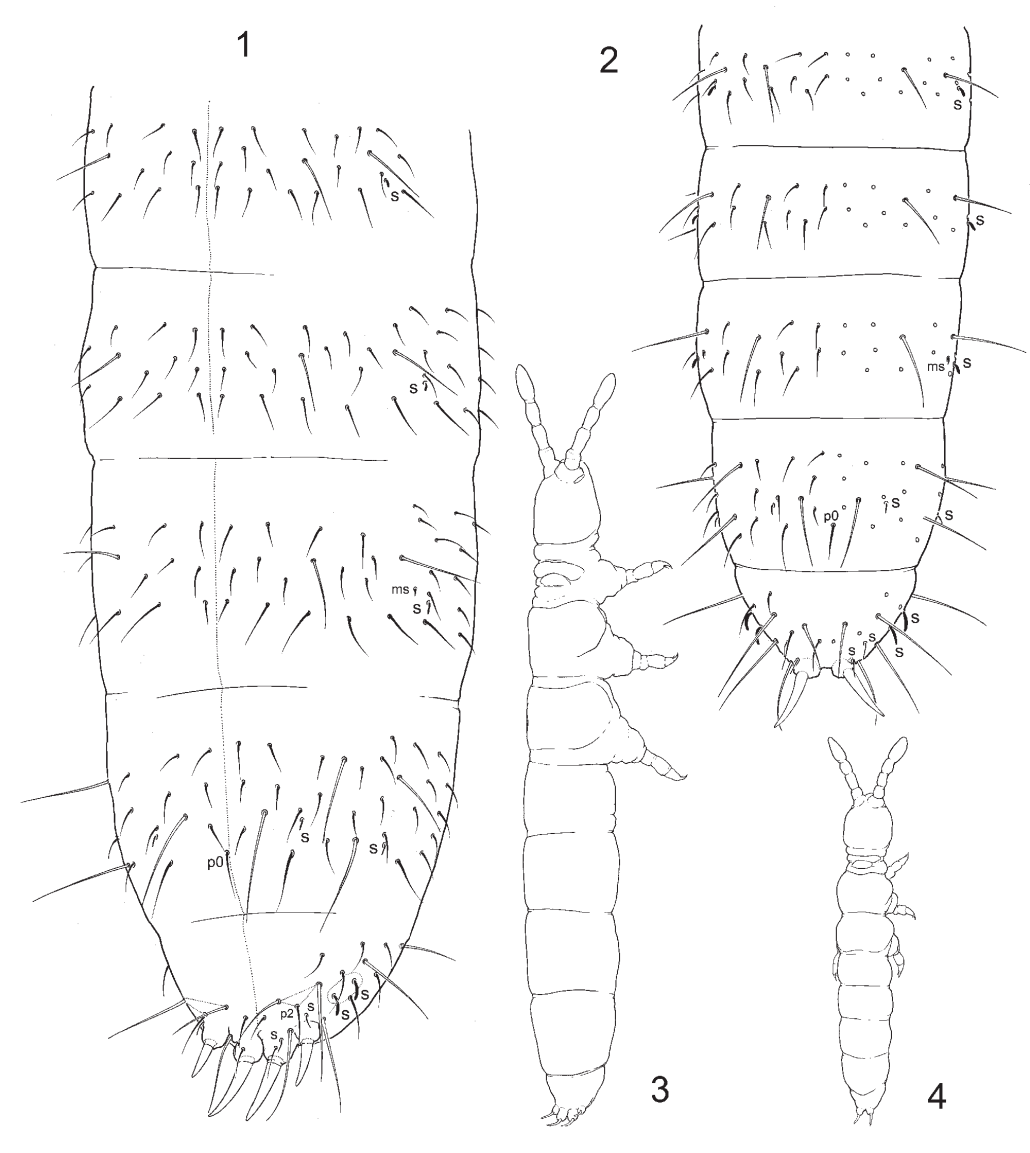
Dimorphacanthella anommatos **1–2** chaetotaxy of abdomen, **3–4** general habitus, **1, 3** adult specimen **2, 4** young juvenile specimen.

Ocelli and pigment absent. Abd. V and Abd. VI fused. Integument regularly and slightly reticulated, without pits. Middle-sized and large individuals with four anal spines set in one transversal row on posterior edge of Abd. V (Figs 1, 10), small juveniles with only two inner anal spines on Abd. V (Fig. 2) (unexamined small juveniles for Dimorphacanthella mediaseta). Inner anal spines of all age stages derived from p1-setae. Setae a1 and p2 not modified to spines. Maxillary outer lobe with simple palp and 4 sublobal hairs. Prelabrum with 2 setae. In two known species labial palp lost five guards on papillae B, D, and E. Body chaetotaxy oligochaetotic. Unpaired median seta (p0) present on Abd. IV. Papilla of inner anal spine is supplied with a seta on dorsal side (absent in small juveniles). Macrosetae well differentiated, macrochaetotaxy of Th. II-Abd. III 1,1/2–3,2–3,2–3. Sensilla well differentiated, 2,1/1,1,1,2,4 (s) and 1,0/0,0,1 (ms) in number. Empodial appendage present, furca absent.

#### Distribution:

China (Shanghai, Ningxia).

#### Type species:

Dimorphacanthella anommatos (Chen & Yin), 1984, comb. n.

#### Remarks:

Sharing four anal spines on genital segment, the new genus Dimorphacanthella formally resembles three genera namely Tetracanthella, Blissia Rusek, 1985 and Sibiracanthella Potapov & Stebaeva, 1995. All four genera also have the same derivation of inner anal spines which are, following the notation of Deharveng (1978), p1-setae modified. Outer anal spines derived from p2-setae in Tetracanthella (Fig. 14) and Blissia and a1-setae in Sibiracanthella. Outer anal spines of Dimorphacanthella are modified setae positioned in posterior part of Abd. V and between two macrosetae pp1 and pp2. Since the designation of setae of Abd. V is not developed enough we notate these setae as setae x on the schemes (Fig. 15). Seta x is a ordinary seta in late instars of Tetracanthella, while in Dimorphacanthella it is modified to a spine at later instars and absent at early instars (Figs 14–16). Seta p2 which is a lateral spine in Tetracanthella remains unmodified in Dimorphacanthella and is located anterior to and between sensilla s1 and s2. The new genus has a pair of ordinary setae in front of median spines in late instars (notated as m1 seta by us) (Figs 15). These setae were never seen in other genera having spines in p1-position. These and other differentiated characters of genera with four anal spines are presented in Table 1.

**Table 1.** Differentiating characters of genera with 4 anal spines on Abd. V and none on Abd. VI

character	Tetracanthella	Blissia	Sibirocanthella	Dimorphocanthella gen. n. (adult)
medial pair of anal spines	p1	p1	p1	p1
lateral pair of anal spines	p2	p2	a1	x
cuticle	slightly to strongly reticulated without pits	slightly reticulated with numerous pits	smooth without pits	slightly reticulated without pits
s formula (ms not considered)	3,3/2,2,2,2,4	3,3/2,2,2,2,3	2–3,2–3/1–2,1–2,1–2,1–2,4	2,1/1,1,1,2,4
seta on anterior side of papilla of medial pair of anal spines (m1)	-	-	-	+
unpaired seta p0 on Abd. IV	-	-	-	+
ocelli	present	present	present	absent

Blindness and presence of unpaired seta on Abd. IV are only shared with Martynovella nana nana (Martynova, 1967) described from Central Asia. This species, however, has two anal spines at all age instars, more complete sensillary set, lacking seta m1 in front of spines, and other less significant differentiating features. Another subspecies, Martynovella nana kirgisica (Martynova, 1967) has no unpaired seta on Abd. IV.

The number and homology of real anal spines are of high value in generic taxonomy of Anurophorinae and appeared to be constant in the course of postembryonic ontogenesis (Deharveng 1978), except epitokous and ecomorphic forms of Proisotoma Börner, 1901 and Cliforga Wray, 1952 (Fjellberg 1988; Najt 1983). In the family, Dimorphacanthella anommatos is the only case where the real anal spines on high papillae appear after moulting.

#### Etymology:

The name of the new genus refers two morphs, juvenile and adult, having different number of anal spines in at least one species of the genus.

### Dimorphacanthella anommatos

(Chen & Yin, 1984) comb. n.

Figs 1 7

Tetracanthella anommatos  Chen & Yin, 1984 (basionym)
Uzelia anommatos  Yue & Yin, 1999, syn. n.

#### Type material.

In the collection Shanghai Insect Museum, holotype and paratypes have not been designated within type series of Tetracanthella anommatos and Uzelia anommatos. Therefore, a lectotype (female) and 2 paralectotypes (females) for Tetracanthella anommatos on slides labelled as “E China, Shanghai City, Songjiang County, Sheshan, 16. III. 1983, Y.M. Yang and B.R. Chen leg.” and a lectotype (juvenile specimen) of Uzelia anommatos on slide labelled “E China, Shanghai City, Shanghai Botanical Garden, bamboo plantation, 5.XII.1997, Q.Y. Yue leg.” were designated by us.

#### Other material.

20 adult and subadult individuals of which 15 are females, one male and four juveniles, E China, Shanghai City, Shanghai Botanical Garden, in Bamboo plantations and under Viburnum macrocephalum, collected in winter time of 1997, 1998 and 2007 by Y.M. Yang and Q.Y. Yue; 1 females, E China, Shanghai City, Shanghai World Exposition Site, litter and soil from 0–15cm depth, 6.IV.2006, Y.X. Luan, Y. Gao and Y. Bu leg.; 4 females, NW China, Ningxia Province, Liupan Mountain Nature Reserve, Longde, Sutai Forest Farm, Sample 7, 35°26'N, 106°11'E; 2145 m alt., grassy slope and forest litter. 21.VI.2008, 1 female, Jingyuan, Erlonghe, Xiaonanchuan Forest Protection Area, 35°23'N, 106°17'E; 2163 m alt., slope beside a ditch, under a rotten tree stump, soft soil. 10.VII.2008, Y. Bu and C.W. Huang leg.

#### Redescription.

Body slender (Fig. 3), 1.0 to 1.3 mm long. Without ocelli and pigment. Body with well visible reticulation, no elongated polygons, the largest ones subequal to sockets of ordinary setae. Maxillary head with strong claw, lamellae not beyond its tip. Maxillary outer lobe with simple maxillary palp and with 4 sublobal hairs. Labral formula as 2/554, edge of labrum without clear papillae, beyond the tips of setae of distal row. Labium with all five papillae (A–E)present. Set of guards incomplete (11) – guards b4, d4, e3 (or e5), e4 and e7 lost. Papilla E with only four guards. Lateral process of labial palp long, at the level with or a little beyond tip of papilla E. Hypostomal setae h1 and h2 long, seta H shorter and thicker, small extra spinule nearby seta H (as in Fig. 9). Proximal part of labium with 3 setae, basomedian field with 4 setae. Ventral side of head with 3+3 postlabial setae. Ant. I normally with 11 setae, 2 bms, dorsal and ventral, and 2 ventral s, one of which curved and 3–4 times longer than another. Ant. II normally with 16–17 setae, 3 bms and one curved s. Ant. III without bms and 5 s, inner pair of sensilla of AO much shorter and set in cuticular groove. Several sensilla on Ant. IV differentiated, subapical organite rod-like, microsensillum twice as long as sensilla.

**Figures 5–7.**
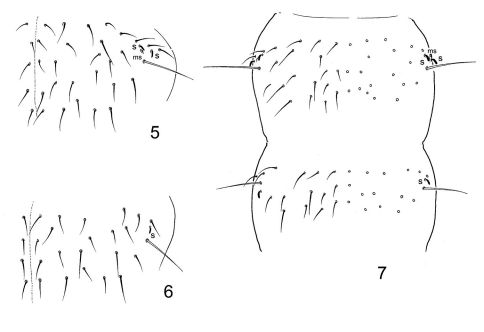
Dimorphacanthella anommatos. **5–6** chaetotaxy of Th. II (5) and Th. III (6) of adult specimen, **7** the same in young juvenile specimen.

Chaetotaxy of adult and juvenile individuals shown on Figs 1, 2, 5–7. Tergal sensilla well visible and thick, much shorter than ordinary setae. Sensillary formula 2,1/1,1,1,2,4 (s). Sensilla almost as wide as ordinary setae, on Abd. V sensilla of lateral pair thick and twice longer than medial one. Microsensillary formula 1,0/0,0,1 (ms). Dorsal axial chaetotaxy of Th. II-Abd. IV as 10(12),8/6,6,6,7. Macrochaetotaxy 1,1/2,2,2,4. Ml macroseta on Th. II 2.7–3.1 as long as p1-seta. Md macrosetae on Abd. IV 1.5–1.9 as long as p0-seta and 2.7–3.2 as long as unguis 3. Sternum of Th. I, II and III with 0+0, 1(2)+1(2), 3(2–4)+3(2–4) setae, respectively. Ti. I–III with 21, 21, 22 setae, respectively. One seta modified (thinner) on inner side of Ti. III. Ventrum of Abd. II with 4 to 5 medial setae, 2(rarely 3) anterior and 2 (rarely 1 or 3) posterior. Retinacular field with 2 (rarely 3) setae. Furcal subcoxa with 9–10 setae, including a pair of macrosetae in posterior position. Manubrial field with 3 pairs of setae. Setulae on anal lobes not seen. Inner anal spines 1.3–1.6 times longer then outer spines. In populations, males much more rare then females.

Age dependent morphology: Small individuals with up to 0.65 mm length have fewer axial setae, 2 postlabial setae, and two anal spines so then corresponding to morphology of Uzelia anommatos. General appearance of fully grown and young individuals is shown in Figs 3–4.

#### Remarks.

For the difference from Dimorphacanthella mediaseta see the remarks section.

### Dimorphacanthella mediaseta sp. n.

urn:lsid:zoobank.org:act:A251FA5A-4412-4C79-9ABA-981113F34163

Figs 8–13 

#### Material.

Holotype: Subadult female with an aperture but not fully developed, body length 0.81mm, NW China, Ningxia Province, Longde County, Shatang Town , Shatang Nanshan Mt. (a little mountain belongs to LiuPan Mountain),  35°23'N, 106°17'E, 1900 m alt., sparse shrubs and birch, 1.VI.2006, Y. Bu, Y. Gao and Y.X. Luan leg.

Paratypes: All from the same area as holotype (Ningxia Province, Liupan Mountain Nature Reserve,), from different localities, all collected by Y. Bu and C.W. Huang. 1 subadult female, body length 0.83mm, same date of Holotype; 1 subadult female, 2 juveniles, Jingyuan, Dongshanpo Forest Farm, Sam 11, 35°37'N, 106°13'E; 2297 m alt., valley, beside a stream, black soil, shrubbery. 24.VI.2008, 1 subadult female, 1 juvenile, Jingyuan, Qiuqianjia Forest Farm, Sam 2; 35°33'N, 106°24'E; 1868 m alt., slope beside a stream, clinosol with many dull leaves, moist soils. 06.VII.2008, 1 subadult female, 4 juveniles, Jingyuan, Woyangchuan Forest Protection Area, 35°39'N, 106°23'E; 1762 m alt., one place is slope, clinosol with dull leaves and humus, another one at the foot of the hill, forest litter, yellow and dry soils with many dull leaves. 29.VI.2008, 3 subadult females, 1 subadult male, 3 juveniles, Longde, Fengtai Forest Farm, 35°35'N, 106°13'E; 2399 m alt., valley, forest litter. 25.VI.2008, 2 juveniles, Longde, Heshangpu Forest Farm (Sam 2; 35°40'N, 106°13'E; 2300 m alt.), valley, forest litter, moist soils. 27.VI.2008, 1 subadult female, Jingyuan, Erlonghe, Xiaonancuan Forest Protection Area, 35°23'N, 106°17'E; 2163 m alt., slope beside a ditch, under a rotten tree stump, crumbly soils. 10.VII.2008, 5 subadult females, 5 males, Jingyuan, Dongshanpo Forest Farm, 35°36'N, 106°15'E; 2125 m alt., foot of cliff, moist soils and moss on the stone. 23.VI.2008.

Holotype and the most paratypes are deposited in Shanghai Institute of Plant Physiology and Ecology, Shanghai Institutes for Biological Sciences, CAS (China), except 3 paratypes are kept in Moscow State Pedagogical University (Russia).

#### Description.

**Figures 8–13.**
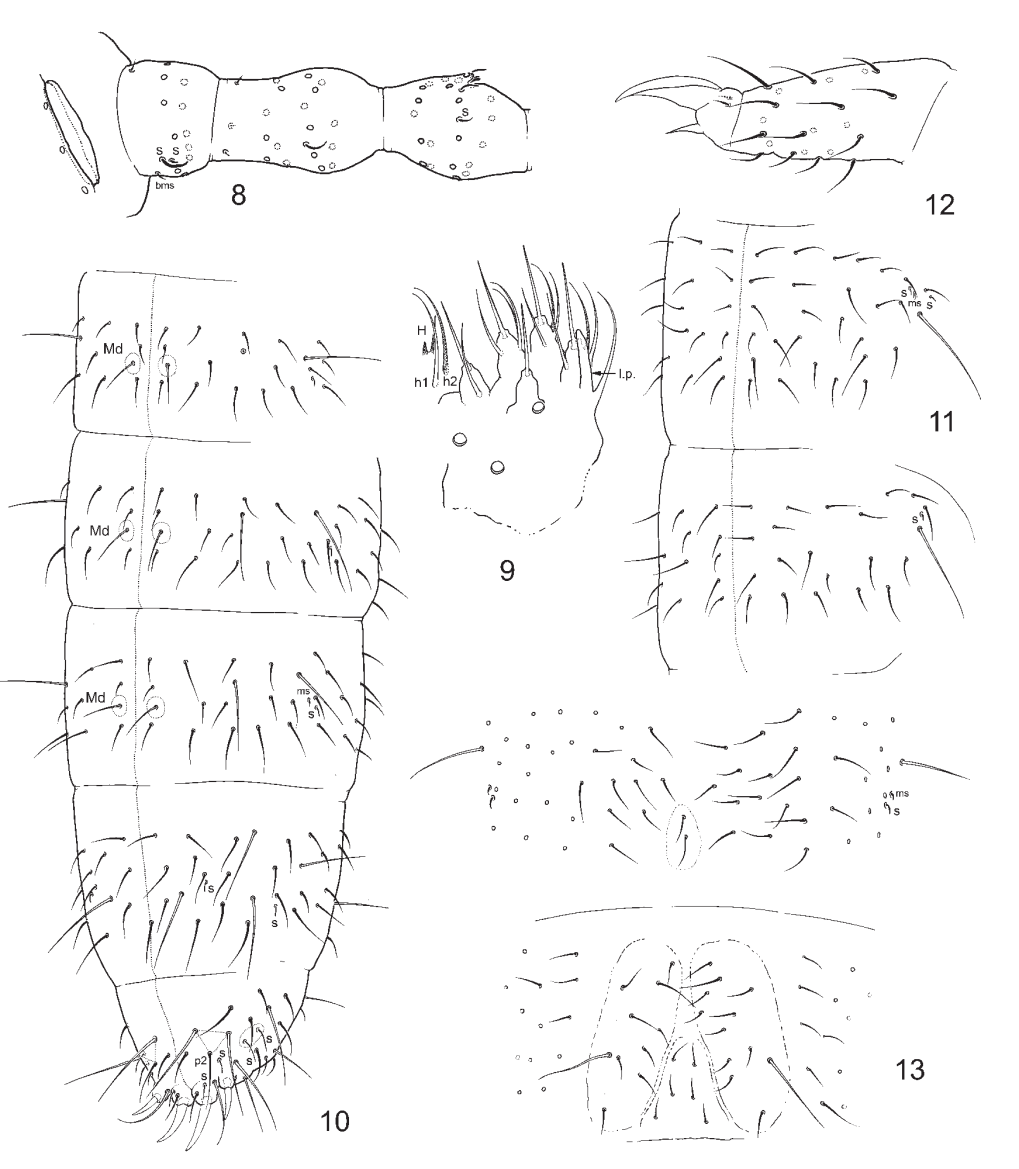
Dimorphacanthella mediaseta sp. n. **8** postantennal organ and three antennal segments **9** labial palp **10–11** chaetotaxy of abdomen (10) and thorax (11) **12** apical part of Leg 3 **13** ventrum of Abd. III and IV. H, h1, h2 - hypostomal setae, l.p. - lateral process.

Size of adult males and subadult females up to 1.0 mm (adult female not seen). Body shape slender, general appearance somewhat that of the genus Stenaphorura (Absolon, 1900) (Onychuridae). No pigmentation. Body with well visible reticulation, no elongated polygons. With 4 strongly chitinized anal spines arranged almost in one transversal row at the end of abdomen. Inner anal spines 1.1–1.4 times longer then outer spines. Ocelli absent. PAO narrowly elliptical, with weak constriction, 1.8–2.2 as long as unguis 3. Outer mouth parts as in previous species, labial palp shown in Fig. 9. Maxillary claw strong, lamellae not beyond its tip. Ventral side of a head with 3+3 postlabial setae. Ant. I with 11 (rarely with 10) setae, 2 bms, dorsal and ventral, and 2 ventral s, one of them curved and 3–4 times longer than another. Ant. II normally with 17 setae, 3 bms and one curved s. Ant. III without bms and with 5 s, inner pair of sensilla of AO much shorter and set in cuticular groove (Fig. 8). Several differentiated sensilla on Ant. IV, subapical organite small, subapical microsensillum short.

Chaetotaxy of body shown on Figs 10–11. Tergal sensilla well visible, much shorter than ordinary setae. Sensillary formula 2,1/1,1,1,2,4 (s). In one individual 2 sensilla on one side of Th. III. Sensilla thinner than ordinary setae, on Abd. V sensilla of lateral pair thin and twice as long as the medial ones. Microsensilla large, set near sensilla, microsensillary formula 1,0/0,0,1 (ms). Tergites with a sparse cover of ordinary setae. Dorsal axial chaetotaxy of Th. II–Abd. IV as 12(14),10(9)/8,8,8,7 (including macrosetae Md). Macrochaetotaxy 1,1/2+1,2+1,2+1,4. In axial group of Abd. I-III a pair of small Md macroseta present (notated as ‘+1’ in formula and encircled on Fig. 10). Short Md macroseta appears to be also present in axial group of Th. II and III but hardly differentiated. Ml macroseta on Th. II 2.6–3.1 as long as p1-seta. Md macroseta on Abd. IV 1.7–2.2 as long as p0-seta and 2.5–3.3 as long as unguis 3. Sternum of Th. I without setae, sternums of Th. II and III with 1+1 and 3(2)+3(2) setae, respectively.

Unguis without inner and lateral teeth. Unguiculus simple, without lamella, 0.4–0.5 as long as unguis 3. Ti. I-III with 21, 21, 22 setae, without additional setae (Fig. 12). Only one seta slightly modified on inner side of Ti. III in both sexes. Tibiotarsal tenent setae weakly developed. Ventral tube with 4+4 laterodistal and 4 posterior setae in one transversal row. Ventrum of Abd. I with four medial setae, two anterior and two posterior. Furca and tenaculum completely absent. Furcal subcoxa with 9–11 setae, of which two longer setae, macrosetae in posterior position and anterior slightly enlarged seta (Fig. 13). Manubrial field normally with 3 pairs of setae. Retinacular field with 2 setae. Each anal lobe with 2 rudimentary setulae, not always distinct. Males present.

**Figures 14–16.**
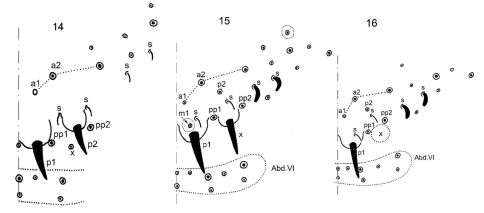
Arrangement of setae, spines and sensilla on dorsal side of Abd. V-VI in Tetracanthella and Dimorphacanthella. **14** Tetracanthella pilosa **15–16** Dimorphacanthella anommatos, adult (15) and young juvenile (16) specimens. In Fig. 15 setae of Abd. V missing in young juveniles are enircled.

#### Remarks.

The new species differs from Dimorphacanthella anommatos by presence of medial pair of macrosetae on the first three abdominal tergites. Sensilla on tergites, especially lateral pair on Abd. V, are thinner than in Dimorphacanthella anommatos. Seta p2 on Abd. V also sharply discriminates these two species (in more posterior position in Dimorphacanthella mediaseta, Figs 1 and 10). Number of anal spines in small juvenile individuals is unknown.

#### Name derivation.

The name refers to medial macrosetae on body differentiating the new species.

#### Barcoding analysis.

DNA barcoding sequence was proved very efficient for characterising Collembolan species (Hogg and Hebert 2004). Here, we used barcode in order to validate those two forms of Dimorphacanthella anommatos are really belong to the same species, in spite of strong morphological differences.

#### Collembolan specimens.

Collembolan species for DNA barcoding analysis were collected from Zhongjia Mountain, 31°05'N, 121°09'E, 25 m alt., Songjiang county, Shanghai city in 75% ethanol by the Tullgren funnel method. They were stored in 100% ethanol at -20°C after morphological identification. We barcoded separately 2 individuals of ‘Tetracanthella anommatos’ with four anal spines and 4 individuals of ‘Uzelia anommatos’ with two anal spines.

#### DNA extraction, amplification and sequencing.

Genomic DNA was extracted from one individual using the Wizard SV Genomic DNA Purification System (# 2361). The mitochondria COI gene sequence was amplified (658 bp) by primer pair LCO (5’ - GGTCAACAAATCATAAAGATATTGG-3’) / HCO (5’– TAAACTTCAGGGTGACCAAAAAATCA–3’) (modified from Folmer et al. 1994). We use the following profile: 94°C initial denaturing for 4 min; 10 cycles of 94°C denaturing for 30 s, 45°C annealing for 30 s, and 72°C extension for 1 min and 30 s; then, with 25 cycles of 94°C denaturing for 30 s, 50°C annealing for 30 s, and 72°C extension for 1 min and 30 s; and a final extension at 72°C for 8 min. PCR products were purified and then were sequenced directly using both of the amplification primers.

#### Results.

Six sequences (length 658 bp) were obtained from 6 individuals. The two individuals of the four-spined form showed the same sequence, as the four individuals of the two-spined form. This suggested that the two forms are a same species in agreement with our morphological result. GenBank Accession number of barcoding sequences are HM366600 and HM366601.

## Supplementary Material

XML Treatment for Dimorphacanthella

XML Treatment for Dimorphacanthella anommatos

XML Treatment for Dimorphacanthella mediaseta
